# Validation of Telehealth Outcome Categories for Patient Safety: Systematic Literature Review

**DOI:** 10.2196/75486

**Published:** 2025-10-16

**Authors:** Sari Palojoki, Lasse Lehtonen, Riikka Vuokko

**Affiliations:** 1 Department for Steering of Healthcare and Social Welfare, Ministry of Social Affairs and Health, PO Box 33, Helsinki, FI-00023, Finland, 358 295 16001; 2 Diagnostic Centre, Hospital District of Helsinki and Uusimaa, Helsinki, Finland

**Keywords:** telehealth, telemedicine, outcome, patient safety, health care, literature review, PRISMA

## Abstract

**Background:**

Integrating telehealth into established care processes can be challenging. With the integration of telehealth into routine health care practices, there is a growing need to evaluate telehealth outcomes to understand its impact on health care delivery. However, existing literature on telehealth outcomes to support evaluation remains limited.

**Objective:**

This study aimed to analyze recent research from the past decade to develop a categorization of telehealth outcomes. This study seeks to validate a defined set of telehealth outcome categories and examine the broader impact of digital transformation on health care delivery.

**Methods:**

We built the telehealth outcome categories according to the existing literature. During the category-building process, we identified 2 main components: the patient safety outcomes of telehealth and the other care-related outcomes. To validate these categories, we conducted a literature search. The initial search yielded 65 unique articles. Following the screening process, we selected 15 articles for the review. In the review analysis, comprehensive data extraction points established a robust framework for evaluating the scope and impact of telehealth research across multiple dimensions.

**Results:**

On the basis of the analysis, 6 patient safety outcome categories were identified: mortality, adverse effects and harm, complications, hospitalization and readmission, diagnostic and treatment errors, and medication safety. The 9 other care-related outcome categories include cost-effectiveness, access to care, and patient satisfaction. Despite a limited sample, the results on the patient safety outcomes of telehealth indicate a generally positive impact. Several studies have reported that telehealth services are associated with reduced adverse events, complications, and readmissions and enhanced monitoring of patient conditions. The reviewed articles did not include use cases covering all identified preliminary outcome categories, such as medication safety and privacy. However, the review supported patient safety categories being well suited to classifying telehealth outcomes. The other care-related outcomes were not so clearly defined and would require more case examples to support category building.

**Conclusions:**

Further refinement of the main categories identified in this article is necessary to allow for the identification of specific areas and themes that warrant further research initiatives. Future research is essential to evaluate the true benefits and outcomes of telemedicine, offering deeper insights into its real-world impact.

## Introduction

The expansion of telehealth signifies a fundamental transformation in the delivery of health care services. The increasing demand for accessible and efficient health care services, especially in light of demographic shifts such as aging populations and rising chronic disease prevalence, as well as a shortage of health care personnel, is among the drivers for the growing implementation of telehealth services. New technological advancements are expected to enhance health care delivery, resulting in more efficient, streamlined, and cohesive care processes [1-3].

Integrating telehealth into existing care processes can be challenging. It is crucial that the transition to telehealth applications is managed carefully to maximize the benefits for all stakeholders, including patients, health care providers, and the broader health care system. Ensuring patient safety is a fundamental priority in health care, and this concern is particularly prominent in the context of telehealth, where the lack of direct physical examinations and in-person interactions can introduce unique challenges and potential risks [3-5].

Despite the potential risks to patient safety, such as miscommunication and preventable harm due to missed or delayed diagnoses, telemedicine has in several studies improved clinical outcomes through timely interventions, reduced mortality, and shortened length of stay [1,4,6-9]. Concerns about the risks associated with telehealth remain, however, since, for example, real-time interactions via telehealth are becoming more common. To date, the literature about the patient safety outcomes of telehealth has not been sufficiently reviewed [4,9]. Although some research exists, comprehensive systematic reviews on alternative modes of delivering telehealth across various care settings remain limited [10].

Although patient safety remains a critical focal point, other outcomes such as clinical efficacy, patient and provider satisfaction, and cost-effectiveness are equally vital in assessing the overall success of telehealth initiatives. With the growing integration of telehealth into routine health care practices, the imperative to thoroughly evaluate these outcomes has become increasingly significant, necessitating a balanced and comprehensive approach to understanding telehealth’s broader impact on health care delivery [11-15].

Given the limited scope of existing studies, a literary review could serve as a foundational step in validating telehealth outcomes. Consequently, this systematic review aims to analyze recent research from the past decade to establish a categorization of telehealth outcomes. By examining studies across diverse settings and populations, it seeks to validate a defined set of telehealth outcome categories and explore the broader impact of digital transformation on health care delivery.

## Methods

### Overview

The rationale for this study lies in the planning phase of an empirical telehealth study. We noticed a lack of comprehensive classifications for telehealth outcomes. Instead, we encountered various reports and studies describing telehealth outcomes and benefits in quite variable ways [eg, 4,16,17]. This led us to compile a categorization of telehealth outcomes based on the existing literature.

Our research question is as follows: Based on the findings of a literature review, can preliminary literature-based telehealth outcome types be systematically built and validated into a categorization?

### Conceptualizing Telehealth Outcome Categories

We began by focusing on the core concepts and their definitions. The World Health Organization (WHO) describes telehealth as “the delivery of health care services, where distance is a critical factor, by all health-care professionals using information and communication technologies for the exchange of valid information for diagnosis, treatment, and prevention of disease and injuries, research and evaluation, and for the continuing education of health-care providers, all in the interests of advancing the health of individuals and their communities” [16].

Telehealth can be defined and understood in multiple ways. According to the WHO, telehealth includes 4 main categories: (1) health care consultations between patients and clinicians, (2) the remote monitoring of patient health or diagnostic data by clinicians, (3) the transmission of medical data to clinicians, and (4) case management consultations between clinicians [2,16]. However, for this review, our analysis is informed by the Organization for Economic Co-operation and Development (OECD) categorization [17], as it provides a more detailed classification of telehealth modalities. The classification emphasizes a technical perspective, distinguishing telemedicine as a subset of telehealth. Here, telehealth encompasses not only clinical services provided remotely but also includes broader applications such as distance learning. However, due to our search objective emphasizing patient safety and other care-related outcomes, specifically distance learning telehealth applications were not included in our review. In summary, in this study, we use telehealth as our primary concept, following the definition provided by the WHO [16], while also acknowledging the OECD’s [17] distinction of telemedicine as a specific subset within the broader telehealth framework.

For patient safety outcomes, we identified the core concepts primarily based on the Agency for Healthcare Research and Quality (AHRQ) patient safety and quality indicators and monitoring tools, which provided a well-established starting point [18,19]. The patient safety and quality indicators are intended to help health care providers assess, for example, the incidence of adverse events and in-hospital complications and identify issues that might need further study. As a general principle, our aim was to create a generic-level category from individual disease-specific indicators, which are sometimes quite precise, to describe the phenomenon at a general level. Based on the AHRQ indicator materials, broad-level patient safety outcome categories were composed preliminarily as follows: adverse events and harm, diagnostic, treatment errors and complications, visits, hospitalization and readmission, and mortality outcomes. Medication safety was added to the categorization due to its overall importance and relevance in patient safety: medication errors occur throughout the medication use process. Harm due to medicines and therapeutic options accounted for nearly 50% of the overall preventable harm in medical care [20].

We also explored several other outcomes associated with telehealth, including patient and provider satisfaction, as well as cost-effectiveness, as defined in the existing literature. For categorizing the other outcomes, as a starting point, we agreed on using the OECD examples for telehealth impacts, which entail the following themes: continuity of care, improved care coordination, and timelines of care; patient-centered care and health literacy; improved quality of care; increased access to care; avoided travel and reduced costs; and increased knowledge sharing and learning [21].

To test and validate the emerging categories, we performed a literary review on telehealth outcomes. For this, we adhered to the PRISMA (Preferred Reporting Items for Systematic Reviews and Meta-Analyses) statement [10,22] to ensure the validity of our review process (Checklist 1).

### Applying the Methodology

The literature search was conducted in July 2024 using the PubMed database, which was selected for its comprehensive coverage of the biomedical literature [23]. PubMed with MEDLINE records enables precise and systematic search strategies using Medical Subject Headings terms [24]. Given PubMed’s extensive repository, no additional databases were queried. The search was documented in detail to allow for reproducibility.

The search strategy (see Textbox 1) was meticulously crafted based on the key concepts of the study: Telehealth serves as our primary concept in line with the WHO definition [[16]], although we also refer to the OECD’s [17] classification. The search was limited to articles published in English and within the past 10 years.

Textbox 1. **Search strategy and filters used.**

((“patient safety” [Title/Abstract]) AND (“telemedicine” [Title/Abstract] OR

“telehealth” [Title/Abstract])) AND (outcome)

Filters: Abstract, Free full text, in the last 10 years, English

The initial search yielded 65 unique articles. In July 2024, the research team initiated the screening process by reviewing the titles and abstracts of all remaining articles. Following the test reading, the researchers convened to discuss and refine the inclusion and exclusion criteria, ensuring a shared understanding. We defined 3 inclusion criteria for the initial screening: an article must document telehealth use case, report patient safety-related outcomes, and be an original research article. During the screening, we identified 14 review articles. The research team decided to process these review articles separately from the original research articles based on the nature of our research questions [2,4,5,10,25-27]. At the same time, we recognized the importance of analyzing the review articles to ensure that we were fully informed by previous research and to support the development of our telehealth outcome categories.

The screening was conducted independently and in a blinded manner, with each researcher applying the criteria separately. Afterward, the results were compared. A match was identified when researchers selected the same option, while a nonmatch occurred if different alternatives were chosen or if a category was not recognized. In 1 borderline case, discussion was required to achieve consensus, but no particularly challenging situations arose. During the initial screening, 42 articles were excluded: 4 of these were due to a lack of telehealth context, 20 due to a lack of patient safety outcomes or other documented health care–related outcomes, and 4 because the paper did not qualify as a research paper (eg, poster).

Ultimately, 23 articles were subjected to a thorough screening process by the authors. For 2 articles, despite being designated as an open access publication, the full text of the article was not accessible. The research team thoroughly reviewed the full texts of the remaining 21 articles to assess their eligibility in July to August 2024. For the final eligibility assessment, the exclusion criteria were defined as follows: the study topic being out of scope or not addressing telehealth outcomes. At this stage, 2 articles were excluded for being out of scope, and an additional 5 articles were excluded for not addressing telehealth-related patient safety outcomes or other health care–related outcomes. Consequently, the eligibility of the identified review articles was also assessed, and 7 previous review articles were included in our study based on their reporting of telehealth outcomes.

The remaining 14 original research articles were systematically analyzed based on specific data extraction criteria following the final inclusion round. One of the included articles described a research protocol where potential result categories were identified. Therefore, our research team agreed to search for an article presenting the results of the study, and this article was included in the review. As a result of this, we had 15 eligible articles for data extraction. The extracted data included the publication year and the journal in which each study was published, providing insight into the academic context of the research. The country of origin of the research and the affiliations of the authors were also documented to assess the geographical distribution.

Each article was further evaluated based on its research goals and motivations, with a focus on the type of telehealth research conducted and whether it pertained to telemedicine or other relevant telehealth innovations. The phase of telehealth intervention within the overall health care process was inspected through 2 categories: prehospital care or posthospital care. Moreover, the clinical domain or specialty, including the specific department involved, was identified to pinpoint the areas of health care most engaged with telehealth solutions. The telehealth application, approach, or method documented in each study was examined to capture the diversity and specificity of the telehealth practices being reported.

Finally, the review recorded patient safety outcomes associated with telehealth interventions, alongside other health care–related outcomes. One of the analyzed articles did not report a specific patient safety outcome despite its research design, instead providing a general discussion on patient safety. Similarly, another article documented patient safety outcomes but did not report other outcomes. After careful consideration and discussion, the research team decided to include both studies due to their valuable content on telehealth outcomes. The content related to these articles is presented in the Results section. These comprehensive data extraction points provided a robust framework for assessing the scope and impact of telehealth research across various dimensions.

## Results

### Overview

**Figure 1.**
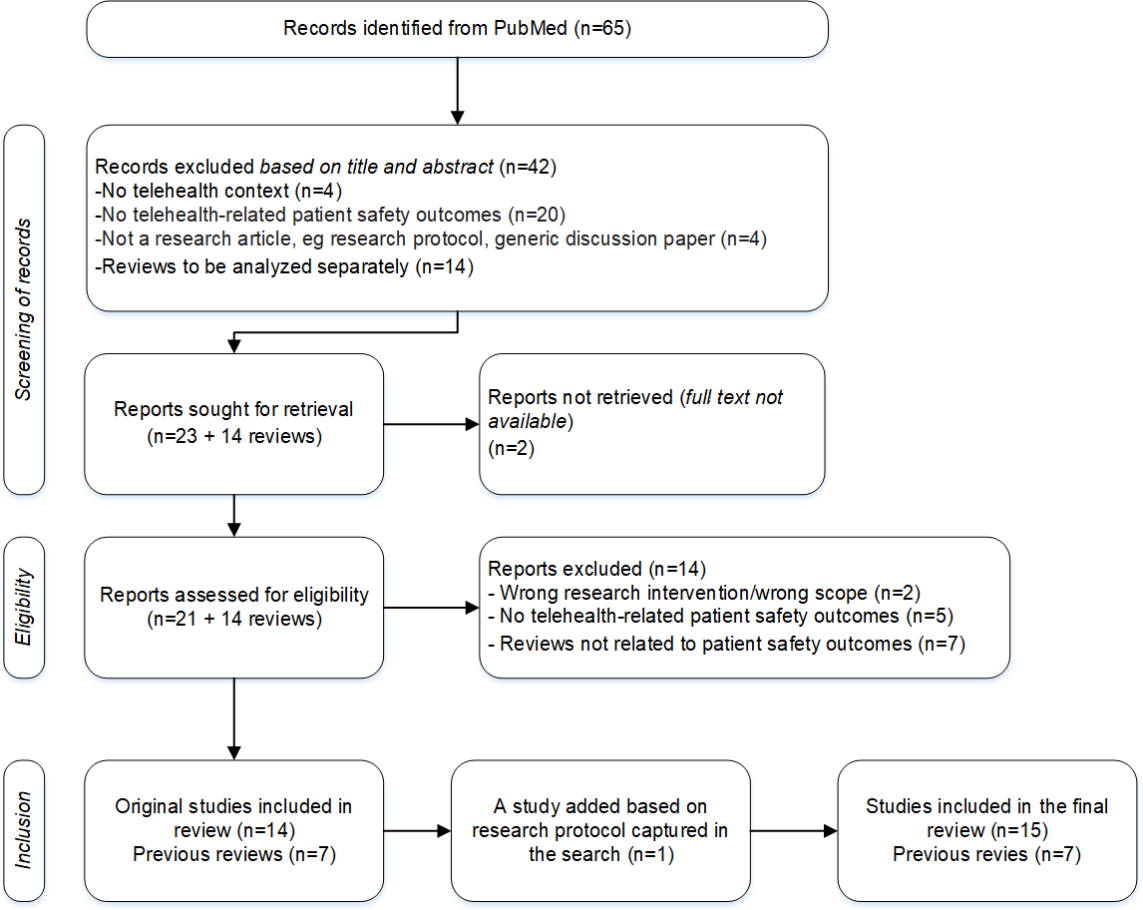
Flowchart of article identification, screening, and final inclusion.

In total, we analyzed the telehealth outcomes from 15 original articles encompassing 14 distinct studies [28-42]. In total, 2 separate publications addressed the same research topic and project but reported different stages of the study. One of the articles was published in 2016, whereas the remaining articles were published between 2019 and 2024. These articles appeared in 14 peer-reviewed journals and 1 National Health Service publication. The studies were conducted across various countries: 3 (20%) in the USA, 1 (6.7%) in both the USA and Italy, 2 (13.3%) in the Netherlands, 2 (13.3%) in Canada, and 1 (6.7%) in both Canada and Argentina. Other countries of origin included Denmark, the United Kingdom, France, the Republic of Korea, Israel, and Singapore. Regarding the research setting, the articles presented a diverse range of studies, including, for example, cohort studies, a feasibility study, and 2 (13.3%) qualitative studies. Notably, 1 of the articles was a study protocol that outlined preliminary outcome categories (Figure 1).

When analyzing the focus of the telehealth intervention within the overall treatment process, it appeared that the research setting in 9 (60%) studies was posthospital [28-31,33,35,39,41,42] and in 6 (40%) was prehospital [32,34,36-38,40]. The clinical setting of the telehealth intervention was described in all studies at a minimum of specifying the patient group involved. These included 2 (13%) studies related to patients with heart failure [41,42], 2 related to high-risk pregnancy monitoring [37,40], and another 2 related to COVID-19, namely to patients with mild COVID-19 symptoms [33] and clinically healthy COVID-19 patients [34]. One study examined pediatric patients [35] and 1 specifically looked at infant patients with congenital heart disease [39]. One study considered patients in emergency care [38], and another considered remote triage of patients with stroke [32]. Other studies included patients undergoing radiotherapy [28], patients with respiratory tract infection [29], patients undergoing bariatric surgery [31], patients with ureteric colic [35], and chronically ill patients at home [30].

Regarding the telehealth approach implemented, several studies described the utilization of 2 or 3 telehealth approaches. Telemonitoring was used in 7 (46%) studies [29,30,34,37,40-42], store and forward telemedicine in 6 (40%) studies [28,30,33,34,36,40], and interactive telemedicine in 11 (73%) studies [28,29,31-39]. Telecare or assisted living at home was not included in any of these studies.

### The Results on Category Building

As outlined in the methods chapter, we developed preliminary categories for telehealth outcomes based on existing categorizations. We used the AHRQ patient safety and quality indicators, along with reported telehealth impact descriptions, as a foundation for developing our telehealth outcome analysis categories [18,21]. To refine the emerging outcome categories, we first enriched them with case examples from the previous review articles identified in our literature search [2,4,5,10,25,26,36] and then standardized the terminology to ensure clearer and more consistent naming.

Our goal was to minimize the overlap between categories. At this stage, in light of the latest research, we decided to add 3 new outcome categories related to clinical outcomes, data protection, and ecological sustainability. The main results of the outcome category building, as used in our review analysis, are presented with references in Multimedia Appendix 1, which contains both key telehealth concepts and the outcome categorization with illustrative examples (on relevant themes in each category) based on previous research. Finally, the telehealth outcome categories are summarized in Textbox 2.

Using the defined set of telehealth outcome categories, we proceeded to analyze the 15 original articles that were included in the review. Detailed results can be found in Multimedia Appendix 2 and Multimedia Appendix 3, as well as in the following 2 result sections.

Textbox 2. **The compiled telehealth outcome categories for our review.**

1. Patient safety outcomes of telehealth
  - Mortality outcomes
  - Adverse effects and harm
  - Complications
  - Hospitalization and readmission
  - Diagnostic and treatment errors
  - Medication safety

1. Other health care–related outcomes of telehealth
  - Clinical outcomes
  - Cost-effectiveness
  - Quality of care
  - Access to care
  - Management and process efficiency
  - Patient satisfaction
  - Provider satisfaction
  - Privacy and confidentiality
  - Ecological sustainability

### The Results on Patient Safety Outcomes of Telehealth

The literature (5/15, 33%) provided references supporting our preliminary categorization of patient safety outcomes related to mortality [29,30,33,37,39]. The analysis of mortality outcomes across the studies suggests that telehealth services may be associated with a reduction in mortality rates for certain diseases. In 1 study, the mortality rate decreased from 16% to 9% [30]. The study on telehealth for infants with congenital heart disease reported no deaths [39], whereas the remaining 3 studies found that telehealth did not result in improved survival or mortality outcomes compared to traditional treatment methods [29,33,37].

The analyzed articles identified an adverse event outcome category that is closely linked to patient safety. Two (13.3%) studies [28,39] reported instances of adverse effects, near misses, or harm associated with telehealth. A study on fully remote radiation oncology care examined patient safety events, defined as staff-reported actual incidents and near misses that could impact patient care [28]. Of the 764 reported safety events, 763 (99.9%) did not reach patients or cause harm to patients, indicating that radiation oncology care provided by fully remote clinicians was safe, with no serious patient events. Another study found no missed events [39].

The reviewed articles supported the complications category, with this outcome identified in 3 (20%) studies [30,37,40]. Our findings suggest that complications among patients receiving telehealth services are relatively rare. In a study on a vital signs telemonitoring system as a regional solution in Italy, the occurrence of major complications decreased from 44% to 30% among the monitored patients [30]. A randomized controlled trial reported a composite of adverse perinatal outcomes as its primary outcome [37]. In the Netherlands, hospitals offering home-based monitoring and telemonitoring for high-risk pregnancies reported improved patient safety as a key outcome of complication monitoring [40]. The category of diagnostic and treatment errors was identified in 3 (20%) studies [32,34,36]. While telehealth improves access to specialists, it may also contribute to misdiagnoses due to limitations in physical examinations and assessments, highlighting the need for enhanced diagnostic protocols [32,34,36].

The analysis of the papers revealed findings that supported our preliminary category of hospitalization and readmission outcomes in 9 (60%) studies [29-31,33,35,37,39-42]. Specifically, these studies suggest that telehealth has an impact on hospitalization and readmission rates. Notably, some studies reported that patients using telehealth services experienced lower readmission rates, likely due to improved management of chronic conditions and enhanced patient monitoring. An Italian study found a 4% reduction in readmissions within 21 days of discharge [30]. Similarly, a study on bariatric surgery patients reported no readmissions at 30/90 days posttelecare [31].

In 1 of the studies, authors do not demonstrate specific patient safety outcomes but that telemedicine used at triage does not compromise maintaining standard care for chest pain and increasing attention to patient comfort via analgesia. Telescreening in triage is both effective and safe, particularly when extending coverage into previously unstaffed hours. No safety concerns specific to telemedicine use (eg, diagnostic errors) were reported [38]. Despite its central importance to patient safety, medication safety is an aspect that was not addressed in the papers reviewed.

### The Results of Other Health Care–Related Outcomes of Telehealth

Other health care–related outcome categories of telehealth were documented in all but 1 of the studies under review (14/15, 93%). Of the 9 outcome categories described in Multimedia Appendix 1 (see also Textbox 2) based on previous telehealth reviews, our reviewed analysis provided research evidence for 8 outcome categories. The category for clinical outcomes [26] was not reported in the studies included in our review, although previous reviews documented examples categorized tentatively as care-related outcomes for telehealth. Feasibility of the tentative outcome category would require more research examples.

The most common other outcome category in our review was cost-effectiveness, which was documented in 11 (73%) of the studies [28,29,31,32,34,37-42]. However, there was no unified way of documenting cost-effectiveness that enables a comparison of telehealth outcomes related to cost reduction. For example, in a telehealth case of radiation oncology, cost savings were calculated per patient [28], whereas another study explored how the intervention costs could be accurately calculated [29]. In this study, clinicians performed telehealth assessments for which average costs were calculated based on the type of contact (telemedicine assessment and domestic visit), number of self-measurements, and severity of alerts. Related to cost-effectiveness, other work-related and occupational benefits were reported [31], such as time saved and no requirement for absence from work to attend a care visit, as well as the possibility to arrange telehealth follow-ups at the workplace. In a telemonitoring case [39], avoidance of emergency department visits contributed to decreasing costs. In addition, indirect cost savings were related to no missed work and no need for transportation. In a COVID-19 telemonitoring case [34], a potential increase in costs and issues of reimbursement were reported, although with no apparent evidence. Another study [38] regarded telehealth costs and a lack of reimbursement as potential barriers to telehealth adoption. Similar reimbursement issues were reported in another case [40]. A high-risk pregnancy telemonitoring case identified patient satisfaction, health-related quality of life, and cost-effectiveness as secondary outcomes of the study [40]. In summary, the results indicate that cost-effectiveness includes many dimensions, especially with regard to telehealth outcomes.

The second most common other telehealth outcome category in our review was patient satisfaction, which was reported in 10 (66.7%) studies [28,29,31,34,35,37-40,42]. In a case of remote management of radiation oncology, nearly all of the patients (n=451, 97.6%) included in the study rated patient satisfaction as good to very good [28]. In a case of a 2-year follow-up after telehealth implementation [31], the satisfaction rate was 80%, with 33% of patients preferring to continue using the telehealth solution. However, 34% of patients felt insecure with telehealth monitoring in relation to health issues that may arise. In a telehealth implementation case [35], 93.1% (n=465) of patients reported satisfaction with the telehealth service. A telescreening case [38] suggested that, in general, patients were happy with telehealth, with a few exceptions. This finding is documented as similar to the evidence gathered from more formal patient satisfaction surveys. Related to patient satisfaction, access to care by remote monitoring was documented as a telehealth outcome only in 1 case [29].

In addition to patient satisfaction, care provider satisfaction was reported as a telehealth outcome in 5 (33%) of the studies [30-32,36,39]. In a case where provider satisfaction was reported, the care personnel were surveyed with 8 different questions to assess their level of satisfaction with the telehealth used in patient care [30]. In an infant congenital heart disease telemonitoring case [39], high levels of provider satisfaction were reported, with physicians expressing an interest in continuing with telemonitoring visits.

The fourth reported health care–related telehealth outcome category was quality of care, which was illustrated in 4 (27%) studies [37,40-42]. In a COVID-19-related telehealth monitoring case [34], the participating professionals reported the possibility to improve the quality of care due to data being measured and shared through the telehealth device. A high-risk pregnancy telemonitoring case [37] reported quality of care related to the advantages of monitoring from home, such as reduced stress and increased rest, including the possible reduction of costs. In a case related to older patients with heart failure [41], home monitoring was expected to improve comfort and quality of life as well as quality of care through the timely detection of possible patient deterioration.

The overall management and process efficiency outcomes related to telehealth were positive, with 4 (26.7%) studies demonstrating improved operational efficiency and patient management [30,34,38,41]. A reduction in the burden on medical staff was documented as a positive outcome [34,36]. Better communication between members of the care team [41] was included in this category. However, the research evidence was partly ambiguous, stating that, for example, higher level patient compliance and workflow optimization would be beneficial [30].

A telehealth outcome categorized as ecological sustainability was documented in 1 of the studies as a result of less traveling by patients and professionals [28]. Similarly, patient privacy and confidentiality were documented in 1 of the studies [30]. One of the original articles [33] reported only patient safety–related outcomes and did not include other health care–related outcomes.

## Discussion

### Principal Findings

Digital transformation and advancements in medical technology, such as remote data exchange and mobile communication, are now reshaping health care. This shift is moving health care from an intermittent, acute care model to one that emphasizes continuous, integrated, and comprehensive care and potentially improving patient outcomes. However, previous research suggests that telehealth outcome measures should be standardized to assess and compare different telehealth implementations’ and use cases’ outcomes more easily [1,10,26]. Despite existing research in this area, an established typology for exploring telehealth outcomes appears to be lacking. Previous research explores telehealth, for example, as a communication means, focusing on its role in facilitating information exchange between patients and health care providers (eg, [11]). Few studies have directly compared different telehealth modalities (eg, [10]). Existing research tends to focus on specific providers or particular conditions [10,25,26,41]. Therefore, we aimed to examine recent research from the past decade to develop an initial categorization for telehealth outcomes.

We built the telehealth outcome categories by using the AHRQ patient safety and quality indicators with an added category for medication safety [18-20] for patient safety outcomes and the OECD telehealth impact categories [21] for other care-related outcomes. During the category building, we checked the overlap between the AHRQ patient safety and quality indicator-based categories and the OECD telehealth impact categories. As the patient safety indicators were described in more detail and were more established, we considered them as guidelines to achieve our data analysis categories for telehealth outcomes. The categories of both groupings were referred to in recent literature reviews, which is why previous reviews were used to further clarify the categories and to refine their naming. Based on a recent review, 3 additional care-related outcome categories were added, namely clinical outcomes, data protection, and ecological sustainability [4,21,25]. Finally, we had 6 outcome categories for patient safety and 9 for other health care–related outcomes (see Multimedia Appendix 1).

The review supported patient safety outcome categories being well suited for classifying telehealth outcomes. However, the medication safety [5,18] category was not met with results in the available study material. Similarly, for other health care–related outcome categories, all were supported with the review except 1 on the clinical outcomes [25]. However, these categories were not so clearly defined originally, and the research team discussed the boundaries and features of the categories to reach a shared interpretation of them. At the same time, the names of the categories were elaborated. Moreover, 2 of the categories were supported with only single examples within the reviewed articles. To summarize, the OECD telehealth impact categories are less clearly defined, being more descriptive and partially parallel to the AHRQ indicators.

On the basis of previous reviews, we decided to introduce new outcome categories related to data protection [4] and ecological sustainability [21]. While no comprehensive validation data were found for these categories in the reviewed literature, their inclusion highlights emerging areas of interest in telemedicine. These categories are particularly relevant, as data protection remains a key concern in health care and ecological sustainability (eg, the need for patient transportation in comparison to digital services) is gaining increasing attention in the context of health care practices [21,28].

The unique nature of telehealth requires patient safety practices specifically tailored to address the risks inherent in virtual care settings. Moreover, the ability to provide safe digital health services anytime and anywhere further underscores the need for robust patient safety practices. Seamless, secure, and universally accessible telehealth services depend on well-integrated safety measures that uphold patient trust while supporting care delivery. In this effort, telehealth outcome data can serve as a starting point for developing virtual care practices, helping to identify and prioritize the most urgent needs for strengthening patient safety measures.

Telehealth outcomes are not always consistent or well integrated, with factors such as device stability and reliability, patient education, accountability, and reimbursement issues impacting the effectiveness of remote patient monitoring [34,40]. While the cost-effectiveness of various telehealth interventions has been studied [28,29,31,32,34,37-42], there are limited data on their long-term efficiency compared to conventional medical practices. In addition, the cost of investment and ongoing maintenance, particularly when multiple stakeholders are involved, may pose significant challenges. Ensuring sustainable implementation requires addressing these financial and logistical barriers while optimizing resource allocation. Future research should focus on evaluating the long-term economic and operational impact of telehealth to guide decision-making and policy development, ultimately enhancing the integration and effectiveness of telehealth solutions.

Our results are significant from multiple perspectives. Our findings have the potential to offer valuable insights for health care providers, policymakers, and researchers, supporting future telemedicine implementations and policies to optimize both safety and other expected outcomes. Moreover, the results could contribute to future telehealth research by providing a systematic approach for classifying and evaluating telehealth outcomes. Structured data on telehealth outcomes could benefit, for example, the assessment of long-term changes in delivering care [26].

By providing a structured approach, these results can shape the future of telehealth, support evidence-based decision-making, and enhance the integration of telehealth into routine care. Furthermore, the study has significant management implications as health care leaders seek to optimize technology resources to improve efficiency. A well-structured telehealth evaluation framework can contribute to ensuring that patient-centered care remains the cornerstone of medical practice, aligning technological advancements with high-quality, accessible, and equitable health care delivery.

Future research is suggested to focus on delineating its scope, establish clear inclusion criteria, and distinguish it from existing outcome categories to improve the reliability and reproducibility of telehealth outcome assessments. Regarding patient safety outcome categories, medication safety warrants further research, given its critical role in ensuring safe care in, for example, preventing adverse events [20]. In the context of building the outcome categorization, it is possible to consider conducting additional, focused literature searches on medication safety–related topics in the next phase of the study. When viewed more broadly from the perspective of telehealth research, future studies should prioritize identifying specific medication safety risks unique to telehealth settings, developing targeted assessment tools, and evaluating intervention strategies to mitigate these risks. To validate “other outcomes” requires also further refinement to enhance conceptual clarity and methodological consistency. In this review, for example, regarding ecological sustainability, because of its emerging nature, it may be necessary to revisit the theoretical foundations concerning conceptualization and factors before proceeding to examine the outcomes.

### Limitations

One limitation of this study is the small number of articles included, which affects the applicability of the findings. More case studies are needed to further validate the results and strengthen the overall evidence base. Incorporating a greater variety of case studies would enhance the robustness of the conclusions drawn. In particular, despite the central importance of medication safety to overall patient safety, this critical aspect was not adequately addressed in the papers reviewed, highlighting a possible gap in the current literature. The same applies to ecological sustainability. Our literature review relied solely on PubMed, which can serve as a primary search tool according to peer-reviewed research [43]. It is, however, essential to acknowledge that the use of a single database may limit the comprehensiveness of the search in areas outside strict biomedicine or in multidisciplinary contexts. While PubMed is the largest biomedical database and citations in PubMed primarily stem from the biomedicine and health fields, and related disciplines such as life sciences, behavioral sciences, chemical sciences, and bioengineering, it has less coverage of fields such as social sciences. For future reviews with broader scope covering also, for example, social sciences, PubMed searches can be complemented with other scientific databases [24,43,44].

### Conclusions

To conclude, preliminary validation based on the literature review supported patient safety outcome categories being well suited for classifying telehealth outcomes. Nevertheless, further refinement of the main categories identified in this article is necessary to allow for the identification of specific areas and themes that warrant further research initiatives. Future research would be critical for assessing the true benefits and outcomes of telemedicine, providing a deeper understanding of its real-world impact.

## Supplementary material

10.2196/75486 Multimedia Appendix 1 Key concepts and outcome categories for data analysis.

10.2196/75486 Multimedia Appendix 2 Characteristics of the publications.

10.2196/75486 Multimedia Appendix 3 Overview of the results.

10.2196/75486 Checklist 1 Preferred Reporting Items for Systematic Reviews and Meta-Analyses (PRISMA) checklist.

## Abbreviations

AHRQ: Agency for Healthcare Research and Quality
OECD: Organization for Economic Co-operation and Development
PRISMA: Preferred Reporting Items for Systematic Reviews and Meta-Analyses
WHO: World Health Organization
