# Supplementary material for: Validation of Telehealth Outcome Categories for Patient Safety: Systematic Literature Review
Source: JMIR Med Inform. 2025 Oct 16;13:e75486. doi: 10.2196/75486 (PMC12530450; doi:10.2196/75486)
Supplement: Multimedia Appendix 1 [file medinform-v13-e75486-s001.docx]

Multimedia Appendix 1: Key concepts and outcome categories for data analysis

| 1. Telehealth    1. The use of ICT to promote health at a distance [16]. Telehealth consists of (1) telemedicine (the use of ICT to deliver health care (clinical services only) at a distance) and (2) telecare (or assisted living; use of ICTs to allow dependent (elderly) to live at home). Telemedicine has three subclasses: (1a) telemonitoring (or remote patient monitoring, tele homecare; use of ICTs to monitor health status at a distance), (1b) store and forward (encounter or consult aided by asynchronous transmission of clinical data) and (1c) interactive telemedicine (or video consultations, real-time teleconsultations, virtual visits; synchronous encounter or consult at a distance using ICTs). [17] 2. Patient safety outcomes of telehealth    1. Mortality outcomes, e.g., reduced mortality rate, patient death, 6-month mortality [5, 10, 18, 25]    2. Adverse effects and harm, e.g., adverse effects, overall harm [4, 18, 25, 36],    3. Complications [2, 4, 18, 26]    4. Hospitalization and readmission, e.g., preventable hospitalization, reduced return visits. reduced readmission, re-attendance rate within 1 month [2, 4, 5, 18, 21, 25]    5. Diagnostic and treatment errors, e.g., inappropriate treatment, delayed care misdiagnosis or delayed diagnosis [4, 18, 21]    6. Medication safety, e.g., preventing medication side effects and medical error [5, 18] 3. Other health care related outcomes of telehealth    1. Clinical Outcomes. Examples: Wound healing (closure or reduction in area), remission rates (e.g., free from head and neck cancer), overall cancer rates. [25]    2. Cost-Effectiveness. Examples: Economic analyses including overall healthcare savings, cost-benefit ratios. [5, 10, 21, 26, 36]    3. Quality of Care. Examples: Enhancement of care capabilities in rural centers, on-site diagnosis and prescription accuracy, provision of remote specialist care, improvements in health-related quality of life. [5, 10, 21, 25]    4. Access to Care. Examples: Reduction in wait times for consultations, shortened visit duration and treatment times, decreased patient transfer times, quicker access to specialists, reduced inpatient days and overall healthcare resource use. [5, 10, 21]    5. Management and process efficiency, e.g., successful management (patients without emergency admission), duplication of services (telehealth followed immediately by an in-person visit), referral rates / triage rates (referred to emergency department, referred to outpatient clinics, hospital admissions), reduced emergency department visits, decrease in hospital length of stay. All contributing to increased continuity of care. [2, 4, 18, 21, 25, 36]    6. Patient Satisfaction. Examples: Levels of patient satisfaction with telehealth services, perceived convenience, and effectiveness, more patient-centered care and improved health literacy. [2, 10, 21, 25, 26, 36]    7. Provider Satisfaction. Examples: Healthcare provider satisfaction with telehealth systems, ease of use, and impact on workflow including clinical information sharing. [21, 26, 36]    8. Privacy and Confidentiality. Examples: Incidents of privacy or confidentiality breaches, compliance with data protection regulations. [4]    9. Ecological sustainability [21] |
| --- |
