# Supplementary material for: Validation of Telehealth Outcome Categories for Patient Safety: Systematic Literature Review
Source: JMIR Med Inform. 2025 Oct 16;13:e75486. doi: 10.2196/75486 (PMC12530450; doi:10.2196/75486)
Supplement: Multimedia Appendix 2 [file medinform-v13-e75486-s002.docx]

Multimedia Appendix 2: Characteristics of the publications

| Reference | Year of publication | Country | Journal |
| --- | --- | --- | --- |
| [28] | 2024 | USA | JAMA Network Open |
| [29] | 2024 | Denmark | Journal of Telemedicine and Telecare |
| [30] | 2023 | Italy, USA | Sensors |
| [31] | 2023 | France | Obesity Surgery |
| [32] | 2022 | UK (NHS) | Health and Social Care Delivery Research |
| [33] | 2021 | Argentina, Canada | Rev Fac Cien Med Univ Nac Cordoba |
| [34] | 2021 | Republic of Korea | JMIR Formative Research |
| [35] | 2021 | Singapore | Ambulatory, Office-based, and Geriatric Urology |
| [36] | 2020 | Israel | BMC Medical Informatics and Decision Making |
| [37] | 2019 | Netherland | BMJ Open |
| [38] | 2019 | USA | JMIR Med Inform |
| [39] | 2022 | USA | Pediatric Cardiology |
| [40] | 2020 | Netherland | JMIR Mhealth Uhealth. |
| [41] | 2016 | Canada | JMIR Res Protoc. |
| [42] | 2021 | Canada | JMIR Formative Research |
