# Supplementary material for: Validation of Telehealth Outcome Categories for Patient Safety: Systematic Literature Review
Source: JMIR Med Inform. 2025 Oct 16;13:e75486. doi: 10.2196/75486 (PMC12530450; doi:10.2196/75486)
Supplement: Multimedia Appendix 3 [file medinform-v13-e75486-s003.docx]

Multimedia Appendix 3: Overview of the results

Telehealth approach categories: 1. tele-medicine, 1a tele-monitoring, 1b store and forward, 1c inter-active telemedicine, 2 telecare.

Patient safety outcome categories:1. mortality outcomes, 2. adverse effect and harm, 3. complications, 4. hospitalization and readmission, 5. diagnostic and treatment errors, 6. medication safety.

Other health care related outcome categories: 1. clinical outcomes, 2. cost effectiveness, 3. quality of care, 4. access to care, 5. management and process efficiency outcomes, 6. patient satisfaction, 7. provider satisfaction, 8. privacy and confidentiality and 9 ecological sustainability.

| Reference | Clinical setting | Pre/post-hospital | Telehealth approach | User groups | Patient safety outcomes | Other health care related outcomes |
| --- | --- | --- | --- | --- | --- | --- |
| 28 | Radiotherapy patients | Post hospital | 1c, 1b | Physician  Patient  Remote care team | 2 | 2  6  9 |
| 29 | Respiratory tract infection patients | Post hospital | 1a, 1c | Clinician  Patients | 1  4 | 2  4  6 |
| 30 | Chronically ill patients | Post hospital | 1a, 1b | Health care professionals | 1  3  4 | 5  7  8 |
| 31 | Bariatric surgery patients | Post hospital | 1c | Physicians  Patients | 4 | 2  6  7 |
| 32 | Stroke patients | Prehospital | 1c | Ambulance clinicians  Stroke physicians | 5 | 2  7 |
| 33 | Covid-19 patients with mild symptoms | Post hospital | 1b, 1c | Attending physician  Medical treating team  Patient | 1  4 | not specified |
| 34 | Clinically healthy Covid-19 patients | Prehospital | 1a, 1b, 1c | Physicians  Nurses | 5 | 2  5  6 |
| 35 | Ureteric colic patients | Post hospital | 1c | Physicians  Patients | 4 | 6 |
| 36 | Pediatric patients | Prehospital | 1b, 1c | Physicians | 5 | 7 |
| 37 | High-risk pregnancy patients | Prehospital | 1a, 1c | Clinicians  Patients | 1  3  4 | 2  3  6 |
| 38 | Emergency care patients | Prehospital | 1c | Physicians | not specified, general approach | 2  5  6 |
| 39 | Infant congenital heart disease patients | Post hospital | 1c | Medical treating team | 1  2  4 | 2  6  7 |
| 40 | High-risk pregnancy patients | Prehospital | 1a, 1b | Physicians  Patients | 3  4 | 2  3  6 |
| 41 | Heart failure patients | Post hospital | 1a | Physicians  Patients | 4 | 2  3  5 |
| 42 | Heart failure patients | Post hospital | 1a | Physicians  Patients | 4 | 2  3  6 |
